# Envisioning Post-pandemic Digital Neurological, Psychiatric and Mental Health Care

**DOI:** 10.3389/fdgth.2021.803315

**Published:** 2021-12-21

**Authors:** Amit Khanna, Graham B. Jones

**Affiliations:** ^1^Neuroscience Global Drug Development, Novartis Pharma AG, Basel, Switzerland; ^2^GDD/TRD Connected Health and Innovation Group, Novartis Pharmaceuticals, East Hanover, NJ, United States; ^3^Clinical and Translational Science Institute, Tufts University Medical Center, Boston, MA, United States

**Keywords:** neurological disease, psychiatry, COVID telehealth, cognitive behavioral therapy, depression, multiple sclerosis, PTSD

## Abstract

The SARS-Cov-2 pandemic placed a dramatic burden on managed healthcare and perhaps nowhere as evident as in neurological and psychiatric disease care. This said, the duration of the pandemic mandated adaptability of the entire care system and the oft-vaunted benefits of telehealth and telemedicine were subjected to deep scrutiny at scale. Positive experiences were reported by both patients and providers from routine check-ups, to use of cognitive behavioral therapy associated with mental disorders, and management of complex diseases such as multiple sclerosis and other neurological and psychiatric conditions. Integration into standard care looks likely in the post pandemic era with many healthcare systems moving to expand reimbursement categories and develop equitable incentive models for developers and providers. In this commentary we share perspective on how the future of care may evolve through hybrid delivery models, and the advent of new therapeutic approaches which can address pain points identified during the pandemic.

## Introduction

The SARS-CoV-2 virus spread rapidly and relentlessly throughout 2020 and into 2021 becoming one of the worst global pandemics on record (1). At the time of authoring this manuscript over 250 million people have been infected resulting in over 5 million deaths globally. Despite the availability of several approved vaccines, herd immunity is yet to be reached in any nation and mutant forms of the virus continue to emerge (2, 3). The past 2 years have had a dramatic impact on all aspects of life and a transformative effect on the healthcare systems worldwide. In early stages of the pandemic access to healthcare services became severely restricted, as COVID related caseloads surged with patients requiring attentive care, isolated environments and the elimination of visitors. All non-emergency services were subjected to triage based down-selection and access to facilities controlled or eliminated for extended periods. Many residential care facilities were closed or quarantined with, in some cases, fatal consequences as the transmission dynamics of the virus, and particularly its vectoring via airborne aerosols were yet to be fully appreciated (4). Over the ensuing months care for patients suffering from myriad chronic conditions was interrupted, modified or deferred as the healthcare systems struggled to cope with emergency demands. Combinations of federal, state, and local guidelines were also imposed to restrict social movements and access to indoor establishments. As a result of rapid disease spread, lockdown imposed social isolation, and economic uncertainties, concern regarding depression and anxiety rates grew among healthcare workers and the general population at large (5). Worse still, patients suffering from chronic neurological conditions reliant on active interaction with healthcare workers witnessed a dramatic impact on their care. For extended periods the only viable option became telemedical and telehealth services conducted remotely either through video conference or telephone lines. Although much heralded as a cornerstone of future medicine, such approaches and sub-categories e-health (electronic) or m-health (mobile) had yet to be tested at global scale during a crisis, providing a critical opportunity to evaluate impact in a world turned upside down by the pandemic (6). As we will outline herein numerous positive outcomes have resulted, ranging from remotely conducted cognitive behavioral therapy to palliative care, and many of the benefits look set to be incorporated into standard care models post-pandemic. Additional, disease specific, concerns have also been highlighted for patients requiring immunomodulators or immunosuppressive therapeutics. The increased likelihood of patients becoming non-adherent or un-medicated for acute neurological conditions, coupled with lack of access to in-person care and added anxiety surrounding the pandemic painted a challenging picture. As we discuss, many pandemic-inspired learnings on use of telehealth and telemedicine in these patient populations will help augment conventional approaches to managed care as systems return to normal (7). What we learn from this experience should help guide us to improved and more resilient healthcare systems globally going forward.

## Pre-pandemic Telehealth for Patients With Neurological and Psychiatric Conditions

The deployment of telehealth services for patients undergoing treatment for neurological and psychiatric conditions dates back to the middle of the last century. Guiana et al. reviewed telepsychiatry conducted 1946–2019 for diagnosis and treatment of major depressive disorder (MDD) noting generally favorable patient satisfaction rates (8). Similarly, Titov et al. surveyed the MindSpot mental health telehealth services deployed in Australia for the period 2013–2019, reporting notable symptom recovery rates for depression and anxiety (59.5 and 59.8%, respectively) and low deterioration rates (1.4% on PHQ-9 and 2.2% on GAD-7 scales) (9). Patient satisfaction rates were also high with enrollees citing ease of access and reduced stigma associated with virtual care. In order to assess the utility of telehealth against conventional in-person approaches, the gold standard of randomized double blind controlled trials is ultimately needed. In one such large-scale randomized controlled trial, Vedaa reported on the positive effects of digital cognitive behavioral therapy on insomnia severity (10).

With this backdrop, there was existing precedent to scale the deployment of telehealth services for patients with neurological and psychiatric conditions. The speed at which the CoV-2 virus spread however placed unprecedented stress on healthcare systems globally, and had a profound impact on these patients both directly and indirectly.

### Interruption in Care for Patients With Neurological Disease

There is general concern that the CoV-2 virus renders persons with compromised immune systems susceptible to additional severity of the disease, and indeed when vaccines finally became available, this group of individuals were typically afforded priority access (11). In the interim, the prescription of medications with immunodepressant effects became a topic of uncertainty for both HCPs and patients. For example, a number of commonly prescribed antidepressant therapeutics are known to elicit immune responses, and the impact on susceptibility to the CoV-2 virus was unclear, heightening anxiety among patients and providers. Mateen assessed the pandemic's impact on HCP practices toward multiple sclerosis treatment in North America (12). reporting patient volume dropped by an average of 79%. A common theme among those surveyed was the accelerated introduction of telemedicine approaches and related practice model changes (13). Likewise, increased pressure on clinic time heightened interest in at home administration of certain parenteral medications (via use of subcutaneous administered autoinjectors instead of IV infusion) (14) and with the potential to verify and monitor adherence rates via use of so called ‘smart' autoinjectors (15).

### COVID-19 Induced Psychiatric Conditions

As the pandemic unfolded the impact on mental health of citizens across the globe became evident. Data compiled from the USA, China, Spain, Italy, Iran, Turkey, Nepal, and Denmark, indicated elevated rates of anxiety, depression, Post-Traumatic Stress Disorder (PTSD), psychological distress, and stress (16). Risk factors included existing chronic/psychiatric illnesses, female gender, unemployment, age (≤ 40 years), and frequent exposure to social media/news relating to the pandemic. A consortium study by the COVID-19 Mental Disorders Collaborators systematically reviewed data on the incidence of major depressive and related disorders globally over the period Jan 2020–2021. SARS-CoV-2 infection rates and reduction in mobility were directly associated with increased incidence of major depressive disorder. The report concludes with the need to prioritize the strengthening of mental health infrastructures (17). Likewise, surveys and assessments conducted in the MENA region mid-pandemic reported psychological impact of the pandemic coupled with increased mental health awareness among adults (18).

These findings highlighted the need to mitigate the effects of COVID-19 on mental health as an international public health priority (9). Additional studies cataloged the rises in depression, anxiety, and stress levels related to the pandemic experienced among vulnerable sub-groups (elderly adults, homeless persons, migrant workers, mentally ill persons and pregnant women) (19). Other reports highlighted the impact of both short and long term physical distancing and the need for early intervention and prevention measures to reduce incidence of mental health impact (20). Based on learnings from other epidemics and natural disasters, depression, PTSD, substance abuse disorders, domestic violence, child abuse, and a range of mental and behavioral disorders are potential concerns that need to be monitored and addressed moving forward (20). Inspired by psychiatric complications reported during the SARS-CoV-1 epidemic of 2002–3, Vindegaard reported on the impact of the CoV-2 pandemic on patients with numerous preexisting psychiatric disorders (21). Overall some 21% reported a reported worsening of symptoms with specific conditions showing considerable impact e.g., 38% increase for eating disorders and 56% for anxiety. Related studies investigating the impact on health care workers also reported increases in depression and depressive symptoms, anxiety levels, psychological distress and worsening of sleep quality (21). Another report on psychiatric and neuropsychiatric presentations among mental health caregivers highlighted the phenomenon of ‘task shifting' where certain aspects of mental health care are delegated to non-specialists as a consequence of staff availability (22).

Another key study reported on the mental health impact on patients who had contracted CoV-2 around the anniversary of the pandemic (23). Studies of electronic health records from over 236,000 patients diagnosed with CoV-2 revealed that approximately 34% presented with psychological or neurological ailments over the following 6 months, and 13% of the patients reporting a first episode (23). For those admitted to intensive care for treatment, the diagnosis rate rose to over 46% (26% reporting a first episode). By far the most prevalent diagnoses were anxiety disorders (17%, rising to 19% for those admitted to intensive care) underscoring the substantial risk of psychiatric morbidity of CoV-2 in addition to its detrimental impact to society at large (23).

As a result of these and other findings, proactive steps were taken by healthcare systems across the globe to introduce and expand telemedicine and telehealth services for those afflicted by the mental health burden. Tailored services introduced included those for depression, stress, anxiety, anger, grief, PTSD, eating disorders, self-harm and suicide ideation (24). These ranged from chat, text, telephone and videoconferencing services to online self-help platforms.

### The Pandemic Inspired New Approaches in Telehealth for Patients With Neurological and Psychiatric Conditions

The deployment of telemedical services for patients with neurological and psychiatric conditions was dramatically escalated as the pandemic evolved. Despite concerns on format, scheduling, privacy and reimbursement, the transition was relatively smooth with numerous reports underscoring patient satisfaction (8, 24–27). In the USA through the period 2020–21 the use of telemedical services for psychiatric care represented the highest component (~50% of all claims) followed by substance abuse disorders (30%), endocrinology and rheumatology (17%), and neurological medicine (13%) (28). Surveys by the American Psychiatric Association indicated that over 80% of physicians saw 75–100% of their patients via telehealth during 2020–2021, with 90% of those patients recording satisfaction with the approach (25). The pandemic also highlighted the importance of peer-peer support in the management of psychiatric illness. Numerous reports documented the worsening of mental health among various populations across the globe being offset by the positive impacts of peer support provided through a variety of mediums, making this a flexible approach (29). Peer groups are also playing a role in the development of new mental health related apps. One example is the Mind Logger platform, which contains a variety of mental health related applets, designed to assess and administer mental health interventions in the general population. This open source platform has end to end encryption, enables restricted access and offers a variety of data collection features. It is currently being deployed for long term assessments in a large-scale, longitudinal mental health study (30). Just as peer groups and user communities have influenced the development of solutions for management of type I diabetes, a similar role in mental health is likely to be impactful. The pandemic also highlighted the critical role that non profit organizations play in mental health by augmenting and informing managed care. Mental health charities across the globe experienced substantial increase in demand over the course of the pandemic (31). These trusted and crucial components of the care ecosystem have also highlighted the potential links between mental health strain and increased online activity during the pandemic, and the problems in effectively policing content shared on social media by peers (32). At the time of writing the pandemic shows only moderate signs of abatement globally, following which we can expect publication of a large corpus of clinical studies which will provide statistically rigid data to inform post pandemic deployment of telehealth in managed care.

### The Pandemic Drove Growth of the Telehealth Mental Health Services Market

Experiences of both HCPs and patients using telehealth during the pandemic was generally positive, and aggregated telehealth services increased by a staggering 4,300% during 2020 (33). This compares to a very respectable growth rate of 53% over 2019 (33), and current estimates suggest that the industry sector is now worth $250 billion (28). Moreover, in the USA alone it is anticipated that some 20% of all Medicare and Medicaid services will be provided through telehealth post-pandemic. Additional shifts toward Hospital at Home (HaH) models is predicted with telehealth services stabilizing at approximately 38X higher than pre-pandemic levels through 2021 (28). In terms of provision for mental health, the management consultancy Accenture reports a dramatic uptick (+43%) in patients preferring virtual care based on data from seven countries (34). Services involving existing providers will develop in the market together with new entrants e.g., AbleTo Inc. who have now developed a suite of virtual cognitive behavioral therapy solutions tailored for payers (35). For long term adoption the issue of provider guidelines and reimbursement will need to evolve along with the need for new regulatory pathways (36, 37) with services codified against new clinical quality measures (eCQMs) (38, 39).

## Implementing Pandemic Inspired Learnings

The CoV-2 pandemic has allowed telehealth and telemedical approaches for the management of neurological diseases and psychiatric illness to be investigated at scale. Largely positive outcomes have emerged, and changes in reimbursement practice coupled with HCP and patient learnings look to establish telemedicine as a permanent service. It remains to be seen what the balance of conventional and technology assisted care will look like post-pandemic but the concept of care at home seems likely to gain a lot of traction. Predicting specific growth opportunities will be difficult until the pandemic wanes globally, together with return to societal social norms and economic stability. However, it seems evident that innovations that allow patients to integrate care beyond the planned episodic clinic visits will be well received and the following scenarios seem likely:

Many providers had reimbursement coverage established for telehealth based psychological counseling services prior to the pandemic. Post pandemic we can expect evidence-based assessments of best practice at global scale similar to that conducted by the UK National Institute for Health and Care Excellence through the Improving Access to Psychological Therapies (IAPT) program (40). The availability of large scale data uncovered during the pandemic will also help fuel HEOR studies and econometrics to guide future deployment. Examination of various mental health sequelae through the lens of the pandemic will provide insight to the most effective approaches to treatment, including necessary cultural adaptations required (27).

Use of remote symptom tracking and home monitoring may become more widespread, as facilities and HCPs recalibrate service provision and technological advances continue to emerge. Some recent examples include the collaboration between Google X and Biogen on sensors and data analytics (41) studies by the Cleveland Clinic on iPad based monitoring for MS patients, (42) and the dreaMS project in Switzerland which involves identification of digital biomarkers for multiple sclerosis using smartphones (43). General models for the integration of telehealth and remote patient monitoring (RPM) have been advanced based on reflections from the pandemic with proposals for “augmented continuous connected care” driven by human inputs (44).

Enhancements in the experience surrounding how patients receive their medications can also be expected. Transportation directly to patient's residences using new logistics services (e.g., Amazon PillPack) will likely expand (45). Learnings from the consumer retail industry might be applied to develop highly personalized approaches e.g., connecting with smart devices and packaging that engage patients through associated apps. The ability to customize drug packaging may also become a differentiator. In a recent study among multiple sclerosis patients, the device used to administer the drug Kesimpta (a “pen” injector) recorded the most positive experience against a number of comparators, suggesting form factor and esthetics can play in important role in patient preferences (46).

Increased interest in care-at-home services is likely to integrate with the telemedicine experience. One possibility could be demand for self-administered versions of medicaments for neurological and psychiatric disorders currently administered under supervision at medical facilities. For example IV infusions require expert assistance as do certain intra-nasally administered drugs (47). Alternatively, subcutaneous formulations might be self-administered via smart auto-injector at home (thereby recording dose adherence), with the assistance of a telehealth consultation where appropriate (14).

Heightened awareness on the importance of scientific communication and education on topics with the potential to impact patient care. For example the impact of autoimmune disease and immunomodulating therapeutics on immune response to viral challenge was a topic of active debate as the pandemic unfolded, and likely influenced vaccination preferences (48–54). Through a combination of telemedical and in-person interactions with providers, education will help patients appropriately prepare for and address future waves of the current pandemic and variants that may follow.

The future of deploying digital technologies in managed care looks bright, and as the pandemic wanes, we will hopefully have an adapted and more widely accessible healthcare system for a broader community. This said, it will be important to conduct studies post-COVID on the impact of technologies globally based on RWE. For example the European RADAR-CNS consortium are conducting research into the use of wearables and smartphones by patients suffering from depression, MS and epilepsy. Interim findings show widespread and increasing use among both patients and HCP's which is clearly having an impact on clinical practice and expected to grow post pandemic (55). It will also be important to assess impact across different groups, including age variants, where the possibility exists for some less digitally savvy patients to feel disenfranchised and to probe if the more digitally fluent generation X-Z groups feel higher affinity to digital centric healthcare. Data from the latter may be especially important as these persons were typically among the final cohorts to be offered vaccinations and their amplified reliance on electronic communications during lockdown may be risk factors for increased depression and suicide rates due to isolation (56–58). Lastly, the role of the biopharmaceutical industry in helping effect change in digital health may be welcomed. Recent reports suggest that public opinion of the industry has enhanced as a result of its role in vaccines development, and the timing may be opportune to help catalyze the public-private partnerships that will be needed to effect change in managed healthcare post-pandemic (59).

## Data Availability Statement

The original contributions presented in the study are included in the article/supplementary material, further inquiries can be directed to the corresponding author.

## Author Contributions

Both authors jointly ideated, created, edited, and approved this perspective.

## Conflict of Interest

AK and GJ are employed by Novartis Pharmaceuticals.

## Publisher's Note

All claims expressed in this article are solely those of the authors and do not necessarily represent those of their affiliated organizations, or those of the publisher, the editors and the reviewers. Any product that may be evaluated in this article, or claim that may be made by its manufacturer, is not guaranteed or endorsed by the publisher.
